# Replay-Based Incremental Learning Framework for Gesture Recognition Overcoming the Time-Varying Characteristics of sEMG Signals

**DOI:** 10.3390/s24227198

**Published:** 2024-11-10

**Authors:** Xingguo Zhang, Tengfei Li, Maoxun Sun, Lei Zhang, Cheng Zhang, Yue Zhang

**Affiliations:** 1 School of Mechanical Engineering, Nantong University, Nantong 226019, China; zhang.xg@ntu.edu.cn (X.Z.);; 2 School of Mechanical Engineering, University of Shanghai for Science and Technology, Shanghai 200093, China

**Keywords:** sEMG, incremental learning, class-increment, DBSCAN, time-varying characteristics

## Abstract

Gesture recognition techniques based on surface electromyography (sEMG) signals face instability problems caused by electrode displacement and the time-varying characteristics of the signals in cross-time applications. This study proposes an incremental learning framework based on densely connected convolutional networks (DenseNet) to capture non-synchronous data features and overcome catastrophic forgetting by constructing replay datasets that store data with different time spans and jointly participate in model training. The results show that, after multiple increments, the framework achieves an average recognition rate of 96.5% from eight subjects, which is significantly better than that of cross-day analysis. The density-based spatial clustering of applications with noise (DBSCAN) algorithm is utilized to select representative samples to update the replayed dataset, achieving a 93.7% recognition rate with fewer samples, which is better than the other three conventional sample selection methods. In addition, a comparison of full dataset training with incremental learning training demonstrates that the framework improves the recognition rate by nearly 1%, exhibits better recognition performance, significantly shortens the training time, reduces the cost of model updating and iteration, and is more suitable for practical applications. This study also investigates the use of the incremental learning of action classes, achieving an average recognition rate of 88.6%, which facilitates the supplementation of action types according to the demand, and further improves the application value of the action pattern recognition technology based on sEMG signals.

## 1. Introduction

Electromyography (EMG) is generated when a series of electrophysiological and electrochemical processes are triggered by the transmission of action potential signals released by neurons to the neuromuscular junction during muscle activation [1], which can reflect the movement information of the neuromuscular system. EMG is recorded and measured by placing electrodes on the surface or inside the muscle, with surface electromyography (sEMG) having the advantage of being non-invasive and easy to carry out [2]. The technology of sEMG-based movement pattern recognition has a wide range of applications in prosthetic control [3], human-computer interaction [4], motion detection [5], and neurorehabilitation [6]. Early research focused on the use of machine learning algorithms, which were based on feature processing was performed by hand to extract feature information representing the motion state from the original signal and to construct a signal feature set to feed into a classifier for gesture recognition [7,8,9,10,11,12,13]. However, there are some limitations of the machine learning methods: (1) feature extraction relies on expert experience, and the selection and quality of the features directly affect the accuracy of recognition; (2) machine learning algorithms have a limited ability to deal with large datasets that are highly nonlinear and complex.

Deep learning is a recognition technique that can automatically extract features through a convolutional neural network (CNN), has a strong nonlinear mapping capability [14], and has a much better performance in the field of sEMG-based action recognition. Xiong et al. [15] proposed a global and local feature fused CNN structure, which fused the global features extracted by Fourier transformation with the local features extracted by a CNN for recognition and obtained an average accuracy of 88.34% on the NinaPro database. Zhang et al. [16] integrated several attention blocks (including channel attention, spatial attention, and temporal attention) into a convolutional layer and improved the extraction of relevant features through weight calibration, achieving an average recognition rate of 91.64% on the NinaPro and Myo datasets. Qureshi et al. [17] used Log-Mel spectrograms from the field of acoustic signal processing to extract features from sEMG signals with 98.31% and 97.97% accuracies in normal and amputee subjects, respectively.

Instead of being satisfied with only discrete action recognition, some other studies have focused on establishing mapping relationships between sEMG signals and continuous variables such as joint angles, force estimation, etc. [18,19]. Lu et al. [20] proposed a framework combining stacked convolution and long short-term memory network (LSTM) models to establish mapping relationships between sEMG signal sequences and multiple joint angles, achieving a correlation coefficient *R*^2^ of 0.9334 for the fitting results. Ma et al. [21] compared the joint angle estimation accuracy of the LSTM model before and after adding high-level temporal features as inputs and proved that the high-level temporal features could improve the accuracy of the model by 8.57%, which demonstrated better performance. Wu et al. [22] proposed a method for finger-press pressure prediction and motion classification using the Manhattan distance method, to eliminate repetitive information between features, combined with the LSTM method, resulting in an accuracy rate of 97.66% for the six pressure patterns.

In summary, previous studies have achieved high accuracy in action recognition and joint angle prediction in one-time acquisition or short time spans. However, there are still some problems in practice, such as the difficulty of maintaining the long-term stability of action recognition in cross-time recognition tasks. The sEMG signal is easily disturbed by external factors such as muscle fatigue [23], electrode displacement [24], and signal time-varying characteristics [25] during long-term use. Among these, electrode displacement and signal time-varying characteristics have a greater impact on the long-term stability of action recognition. Hargrove et al. [26] conducted several acquisition experiments to simulate the displacement of the sensors in actual use and showed that, in the presence of electrode displacement, the error in the model identification results was 30%. The traditional approach to overcoming this undesirable effect is to collect and store all relevant datasets. However, this imposes a huge memory overhead and affects the real-world application of this method.

Incremental learning is a method capable of learning continuous tasks that change over time [27], and the main application scenarios for this method are task incremental learning, domain incremental learning, and class incremental learning. Through fast training with new data on pre-trained models, high accuracy can be achieved on both new and old datasets. Currently, incremental learning is mainly used in the field of action recognition for the incremental learning of action classes. Hua et al. [28] proposed a spectrum-based early and late fusion convolutional neural network (ELFCNN) architecture and incremental learning framework to enable the classifier to gradually learn different sets of gestures without catastrophic forgetting to cope with the increasing demand of gestures in applications. This study focuses on the intertemporal task, where the sEMG signal exhibits instability over time, and uses incremental learning to overcome this problem.

For the scenario of cross-time gesture recognition based on sEMG signals, the aim of this study is to investigate the effects of electrode displacements and time-varying characteristics of the signals on gesture recognition, and to use an incremental learning approach to learn the non-synchronous data in the cross-time recognition task, in order to overcome the undesirable effects of electrode displacements and time-varying characteristics and to achieve incremental learning of the task and incremental learning of the class. The main contributions of this work can be summarized as follows.

(1) We proposed a gesture recognition framework combining densely connected convolutional networks (DenseNet) and replay-based incremental learning, and which maintains long-term stability in cross-time recognition tasks through incremental learning at a low cost;
(2) The use of the density-based spatial clustering of applications with noise (DBSCAN) clustering algorithm to select samples for replay dataset updating is proposed, which outperforms conventional methods;
(3) Class incremental learning is performed in the cross-time task, allowing the model to learn additional gesture actions.

This paper is organized as follows: Section 2 describes the sEMG cross-time acquisition experiment and the pre-processing method; Section 3 describes the DenseNet model and the replay-based incremental learning method; Section 4 describes the experimental results; and Section 5 and Section 6 are the discussion and conclusion sections.

## 2. Experiment and Pretreatment

### 2.1. Subjects

Eight healthy adult male volunteers were recruited for the experiment (ages 20–28, average weight 79.8 kg), none of whom had a history of musculoskeletal disorders. The volunteers did not perform any strenuous exercise for 24 h before the experiment to ensure that the data were reliable. The volunteers were informed about the experiment and signed an informed consent form.

### 2.2. Signal Acquisition

**Figure 1:**
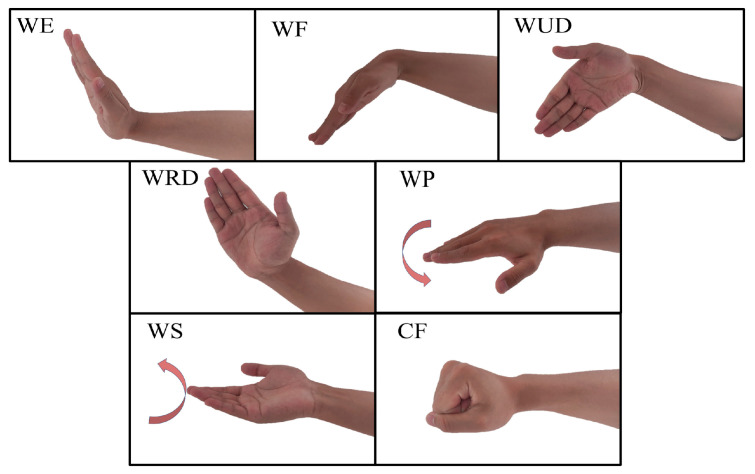
Seven classes of gestures.

**Figure 2:**
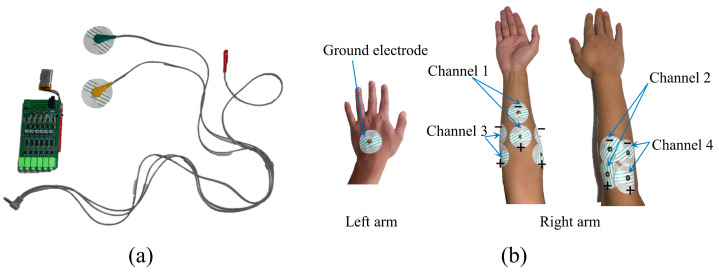
(**a**) Experimental acquisition equipment. (**b**) Electrode attachment position.

The experiment was designed with seven classes of common gestures, including wrist extension (WE), wrist flexion (WF), wrist radial deviation (WRD), wrist ulnar deviation (WUD), wrist supination (WS), wrist pronation (WP), and clenched fist (CF) [29], as shown in Figure 1. The sEMG signal acquisition device consisted of an EMG muscle sensor module (EMG06, Wuxi Boruiyin Technology Co., Ltd., Wuxi, China) and bipolar Ag/AgCl electrodes, as shown in Figure 2a. The sampling frequency of the experimental equipment was 1000 Hz. In the experiment, four-channel sEMG signals were collected from the right arm, and the electrodes were placed on muscle groups closely related to the hand gestures, namely the radial carpal flexor, brachioradialis, ulnar carpal flexor, and radial carpal extensor [24], as shown in Figure 2b, with a ground electrode placed on the back of the left hand. Before applying the electrodes, the subject’s skin was cleaned with an alcohol wipe to ensure that the electrodes adhered and that accuracy of the signal acquisition was achieved.

This experiment adopted a continuous multi-day data acquisition strategy to simulate a cross-time recognition task. Each subject performed one experiment per day. In each acquisition experiment, each type of gesture was performed 100 times with a 10 min break between each action. The experiments lasted for 12 days, and each subject acquired a total of 12 corresponding non-identically distributed datasets, named with the number of days of acquisition.

### 2.3. Signal Preprocessing

Signal processing consists of two main operations: filtering and action segmentation. In the sEMG acquisition experiments, the greatest influence on the effective information of the sEMG is exerted by Gaussian white noise (WGN) and power line noise (PLN) [30]. Among them, the main effective information of sEMG is concentrated in the 20–300 Hz range, and the filtering part of this study mainly uses a 20–300 Hz band-pass filter and 50 Hz band-stop filter to filter the sEMG signal.

**Figure 3:**
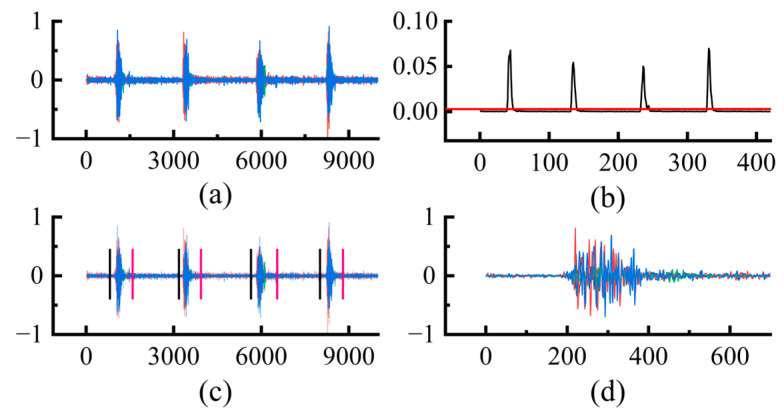
Schematic diagram of action segmentation: (**a**) Filtered signal. (**b**) Calculated average energy map. (**c**) Signal segmentation. (**d**) Individual segmented signals.

Action segmentation means that the signal segment corresponding to each action is extracted from the data as an input to the model, where the key point is to determine the start and end points of the action. In this study, the energy-based thresholding method is adopted for action segmentation [31]. The main steps are as follows:

1. The moving window is utilized to divide the signal into data blocks of equal size;
2. The average energy of the four channels of the data block is calculated according to Equation (1) [31];
3. The start and end positions of the motion are determined according to the set threshold in the average energy graph.
  (1) $$E_{w}\left(t\right)=\frac{1}{4}\sum_{i=1}^{4}\frac{1}{N}\sum_{n=0}^{N-1}{\left|x_{i}\left[n\right]\right|}^{2}$$
  where x is the signal, t is the data block index, and i is the signal channel index. The action segmentation process is shown in Figure 3. The effective length of the extracted action segments varies, and the length of the signal segments is uniformly set to 1000 samples to facilitate input to the CNN model.

## 3. Methodology

### 3.1. DenseNet

Convolutional neural networks have been widely used in the field of sEMG-based gesture recognition, in which the convolutional layer is the key to the convolutional neural network’s feature extraction of the input feature matrix. The transformation between the input and output of the convolutional layer is as follows:

(2) $$X_{i+1}=F_{i}\left(X_{i}\right)$$

where $F_{i}\left(\cdot \right)$ represents the nonlinear transformation, which is a composite function of the operations of the batch normalization (BN), the rectified linear units (ReLU), the pooling layer, and the convolutional layer.

**Figure 4:**
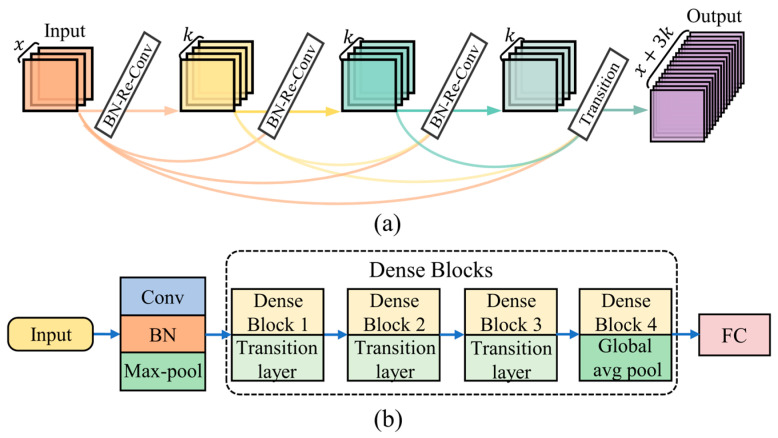
(**a**) A 4-layer dense block with a growth rate of *k* = 4. (**b**) The complete framework of the DenseNet model.

For convolutional neural networks, each layer of the CNN is a feature extractor, so the depth of the network is closely related to the parameters and performance of the model. If the number of network layers is too shallow, the feature extraction and generalization capabilities of the model will be poor. The conventional approach is to improve the model performance by increasing the number of network layers. However, as the number of network layers increases, the problems of gradient vanishing and degradation gradually come to the fore, leading to saturation and rapid degradation of the model’s accuracy after a certain depth threshold is reached [32]. To address these issues, DenseNet [33] introduces direct connections from each layer to all subsequent layers to achieve dense connectivity, which mitigates the gradient vanishing and degradation problems by bypassing the nonlinear transformations, as shown in Equation (3).

(3) $$X_{i+1}=F_{i}\left(\left[X_{0},X_{1},\ldots X_{i}\right]\right)$$

where $F_{i}\left(\cdot \right)$ represents the nonlinear transformation and $X_{i}$ is the output of the i-th layer. The dense connection block structure is shown in Figure 4a. The complete framework of the DenseNet model used in this study is shown in Figure 4b. The structure of the model consists of 3 parts: the feature extraction layer, dense blocks layer, and classification layer. The dense blocks layer mainly consists of 4 dense blocks and each dense block is followed by a transition layer. Among them, the transition layer consists of a BN layer, ReLU activation, 1 × 1 convolution, and an average pooling layer. The classification layer is input after the last dense block through the global average pooling layer. The DenseNet model used in this study has a dense blocks structure of 6, 6, 6, 6, and the growth rate is set to 24. In order to adapt to the 1-dimensional data input, the parameters of the model’s convolution layer and pooling layer are set to be 1-dimensional.

### 3.2. Incremental Learning

The common incremental learning methods fall into two main categories: regularization-based methods and replay-based methods [34]. Regularization-based methods balance model plasticity and stability by introducing regularization terms to constrain the updating of model parameters [35]. However, this approach requires the selection of appropriate regularization parameters and a lot of tuning work, while the introduction of regularization terms increases the complexity of the objective function.

**Figure 5:**
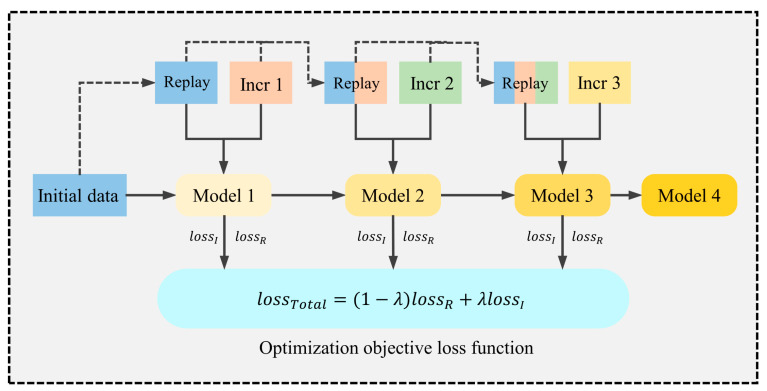
Diagram of the replay-based incremental learning framework.

**Figure 6:**
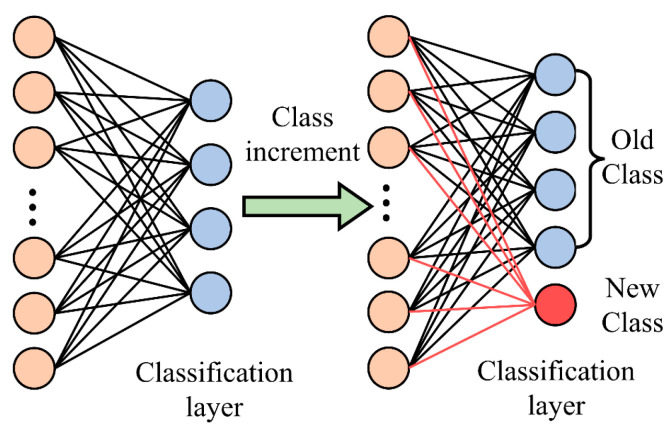
Class increment mechanism diagram.

Replay-based methods help models not to forget old knowledge when learning new data by retaining and reusing some of the old data [28]. This method is simpler to implement than the regularization method, but it requires the storage of some of the old data, which can lead to higher storage costs. Table 1 shows the application of incremental learning in some research areas in recent years. In this study, the input to the CNN model differs from that for traditional image recognition approaches, as it utilizes one-dimensional data signals. This format offers a lower storage cost, which is why the study adopts a replay-based incremental learning approach. The framework of replay-based incremental learning is shown in Figure 5, which demonstrates the mechanism of triple incremental learning. Model 1 represents the initial model obtained by training on the initial dataset. After inputting the incremental dataset, the replay dataset, which stores the old data, and the incremental dataset are jointly input to model 1 for training to obtain model 2, and so on. Each time new incremental data are introduced, the replayed dataset is reconstructed and updated accordingly. Figure 6 shows the mechanism of class incremental learning in deep learning. It adds new classes to the output of the classification layer and links them to the hidden layer.

In deep learning, the loss function is used to measure the difference between the model predictions and the actual labels. The loss function helps to optimize the model parameters so that the model predictions are as close as possible to the actual values. The cross-entropy loss is often used in classification problems, especially multi-class classification problems. The formula for the cross-entropy loss is shown in Equation (4):

(4) $$loss=-\frac{1}{N}\sum_{i=1}^{N}\sum_{c=1}^{C}y_{i,c}\log({\hat{y}}_{i,c})$$

where N is the number of samples, C is the number of categories, $y_{i,c}$ is the actual label of the i-th sample in category c, and ${\hat{y}}_{i,c}$ is the predicted label of the i-th sample in category c.

The replay-based method has two independent loss terms: one for data in the current context, denoted as $loss_{I}$, and the other for replayed data, denoted as $loss_{R}$. During training, the goal is to optimize the overall loss function, which is a weighted sum of these two terms, whose weights depend on the number of contexts seen so far. The overall loss function is calculated as:

(5) $$\lambda =\frac{N_{Incl}}{N_{Total}}$$

(6) $$loss_{T}=\left(1-\lambda \right)loss_{R}+\lambda loss_{I}$$

where λ is the weight given to the incremental dataset, $N_{Incl}$ is the sample size of the incremental dataset, and $N_{Total}$ is the sample size of the full dataset. In the cross-time incremental learning scenario, the minimum value of λ is restricted to 0.2 because the new data set has higher validity. In the class incremental scenario, by default, the old classes have already achieved good accuracy and the input incremental dataset has only new class actions. In this case, λ should be set to the ratio of the number of classes, which in this study is set to 1/7.

### 3.3. Replay Sample Selection Methods

In replay-based incremental learning methods, the quality of the replay dataset is directly related to the model’s recognition performance on the old task, so how to select representative samples from the old task is key to the problem. To better characterize the sample data, this study compares four sample selection methods, including DBSCAN clustering.

Method 1 is the DBSCAN clustering method [38]. DBSCAN clustering is a density-based clustering algorithm that finds regions which are tightly clustered together as well as those regions with a low density of data points. Before using the clustering algorithm for classification, root mean square (RMS) features are calculated for each channel of the action segment. The calculation formula is shown in Equation (7).

(7) $$RMS=\sqrt{\frac{1}{N}\sum_{i=1}^{N}{x_{i}}^{2}}$$

where N is the length of the signal segment, and $x_{i}$ is the signal value at point i.

**Figure 7:**
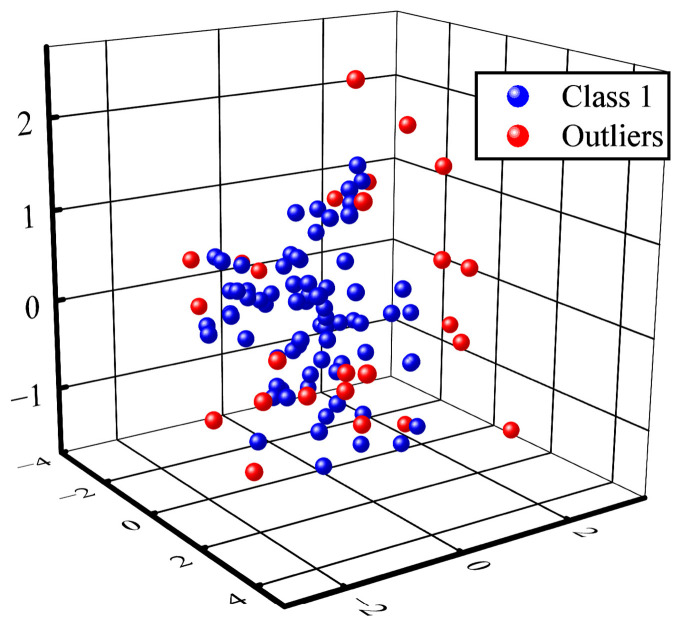
The clustering of samples with DBSCAN, where blue points are similar and red points are outliers.

For each action class, a clustering analysis was performed using the DBSCAN algorithm to filter out the noise points and assess the strength of representation of the action segments based on their clustering distance from the core point. The DBSCAN clustering results after principal component analysis (PCA) downscaling to 3D space was conducted are shown in Figure 7.

Method 2 is the random method, characterized by randomly selecting data samples from the dataset of the old task.

Method 3 is uniform selection. Due to the effects of muscle fatigue, there will be subtle variations in movements performed consecutively. Selecting samples evenly from older datasets can capture these variations more fully.

Method 4 is intermediate selection. This method assumes that there are a certain number of transitional data segments during the execution of the action, and it is assumed that the samples in the center part of each experiment are the most representative [29].

## 4. Results

This study used a four-channel sEMG signal dataset from eight subjects collected over 12 days. Twenty percent of the training set was randomly divided into a validation set to prevent overfitting. In this study, all experimental results are based on the independent training of subjects. The experimental platform used in this study is an i5-13490F CPU@ 4.80 GHz (Intel Corporation, Santa Clara, CA, USA), with a GeForce RTX 3060ti GPU (NVIDIA Corporation, Santa Clara, CA, USA), and 32 GB RAM (Kingbank, Shenzhen, China). The number of training iterations was set to 50, the learning rate was set to 0.001, and the batch size was 128.

### 4.1. Comparison of Single-Day and Cross-Day Analysis

**Figure 8:**
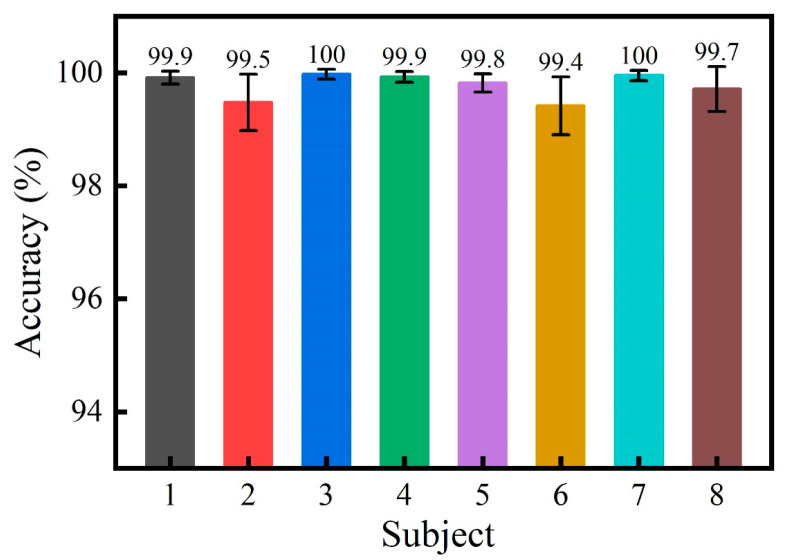
*K*-fold cross-validation results for single-day dataset of eight subjects.

To demonstrate the effect of electrode displacement and time-varying properties on gesture recognition in cross-time scenarios, single-day and cross-day analyses of the dataset were performed and compared. The single-day dataset was analyzed by *k*-fold cross-validation, where *k* = 10. The *k*-fold cross-validation results are shown in Figure 8. The results show that good classification results were achieved using the DenseNet model on the eight subjects in the single-day data analysis, with average recognition rates exceeding 99% on all subjects.

Next, cross-day data analysis was performed. This was done by using a dataset from one day as a training set to train the model and then testing it on datasets from other days. This approach introduces a time span that can reflect the effect of electrode displacement and time-varying characteristics of the signal on the detection results. A summary of the accuracy of the cross-day analysis is shown in Table 2. The horizontal column of the table indicates the number of days in the training set and the vertical column indicates the number of days in the validation set. The results show that the recognition rate of the model in the cross-day analysis shows a significant decrease to varying degrees. After aggregation, the overall recognition rate is 64.2%, which is a decrease of almost 35.6% compared to the single-day analysis.

**Figure 9:**
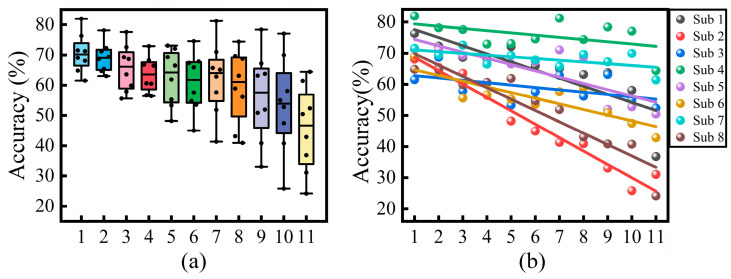
(**a**) Box plot of accuracy across days. (**b**) Plot of the linear fit of recognition rate over time span, where the horizontal coordinate indicates the time span.

The trend of each subject’s model recognition rate over the time span is illustrated in Figure 9a. The results show a decreasing trend in model accuracy as the time span increases. The highest average identification rate of 70.6% was achieved at a time interval of 1 day, which is almost 29.2% less than for the single-day analysis. At this point, due to the small time interval, the error is mainly caused by electrode displacement. The lowest average recognition rate was found at 11 days, with 45.5% accuracy, which is 25.1% less than the one-day interval. In this case, the error is influenced by the combination of electrode displacement and temporal features. Also looking at the error of the box plots, it was found that the variance in the recognition rate increased with the increase in the time span, showing a significant instability at a time span of 11 days.

The results of different subjects after linear fitting are shown in Figure 9b, which shows that different subjects have different sensitivities to the variations brought about by the time-varying properties. Among them, subject 2 is the most sensitive to time, with a slope of −0.0429 and the fastest decline, and subject 7 is the least sensitive and had the slowest decline, with a slope of −0.0056.

### 4.2. Incremental Learning with Multi-Day Data

In multi-day data incremental learning, the order of the application of the dataset increment strategies is a key factor. The results of the cross-day analysis show that, in practical applications, the validity of the data that are initially acquired gradually decreases as the time span increases, which leads to a decrease in the performance of the models trained on these data. For this reason, models’ performance could be improved by introducing data with a shorter time span and higher validity for retraining.

To simulate this process, after determining the test set, this study adopts the method of sorting by time span to select the initial training set and the incremental training set. Specifically, the two datasets with the largest time span were used as the initial training sets, and then the datasets with smaller time spans were added incrementally in order. Then, the cyclic operation was performed for different test sets. The specific training strategy is shown in Table 3.

**Figure 10:**
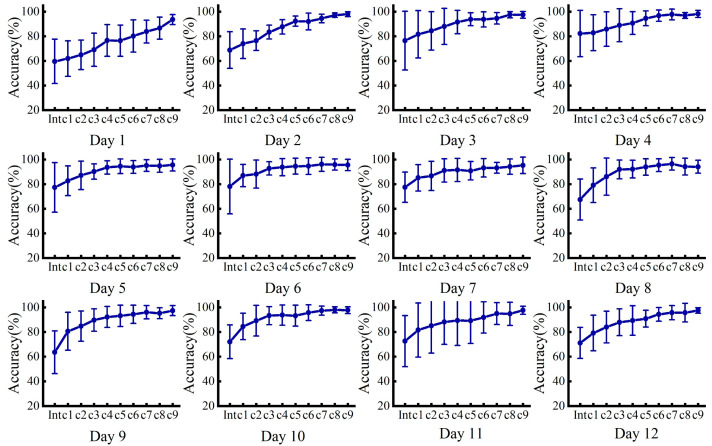
Incremental learning results under different days’ test sets, where the different subplots indicate the number of days in the test set.

Figure 10 illustrates the incremental learning results under different days’ test sets. The results show that, with the input of the incremental dataset, the recognition rate of the model shows an upward trend, while the variance decreases and the model performance becomes more stable. After nine increments, the model achieves an average recognition rate of 96.5%, which far exceeds the recognition rate for the cross-day data. Depending on the test set and training strategy, the results show a more obvious upward trend in the case of a larger time span between the initial training set and the test set, such as when the test sets are from 1 day and 12 days, with a lower initial recognition rate but a larger enhancement, which is more representative. In the case of a smaller time span between the initial training set and the test set, such as when the test set is from 6 and 7 days, the recognition rate still shows an increasing trend, but the initial recognition rate is higher and the overall recognition rate is also higher and more stable.

In practice, incremental datasets are usually not available for multi-sample sampling, and we therefore tested these in the few-sample case as well. In this case, the initial training set is unchanged and the incremental dataset is set to 10 and 20 samples per action, respectively, obtained by uniform extraction from single-day data. The size of the replay dataset was also reduced proportionally. In the results, we only use representative 1-day and 12-day sets as the test sets for comparison.

**Figure 11:**
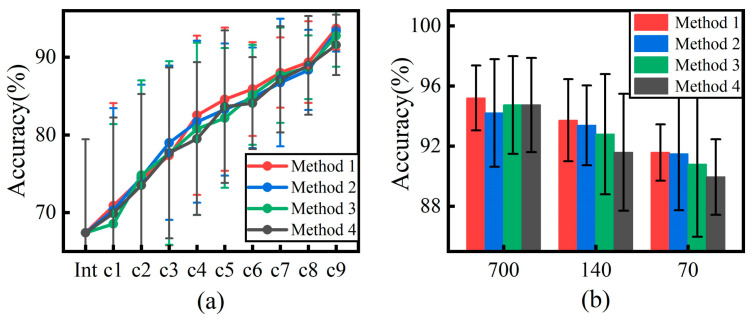
(**a**) Comparison of the four data selection methods with fewer samples (**b**) Comparison of the accuracy of the four methods with different sample sizes.

The incremental learning results of the four methods in the case of the use of 140 incremental samples are shown in Figure 11a, where the horizontal coordinates represent different incremental sample sizes. Overall, the accuracies of the four methods are comparable and there is no significant difference. However, in the second half of the incremental process, the clustering method shows better performance than the other methods due to the increase in the number of increments, the increase in the amount of old task data, and the decrease in the amount of data extracted from the daily dataset. Figure 11b shows the results of the ninth increment for the four methods with different incremental samples. The results show that, as the number of incremental samples decreases, the recognition rate of the model shows a decreasing trend. Meanwhile, the four data selection methods show performance differences in the small-sample case. Among them, the method using cluster selection performs the best, with recognition rates of 93.7% and 91.6% in the cases of 140 incremental samples and 70 incremental samples, respectively. In contrast, the method using intermediate selection had the lowest recognition rate, with 91.6% and 90.0% in the small-sample case.

**Figure 12:**
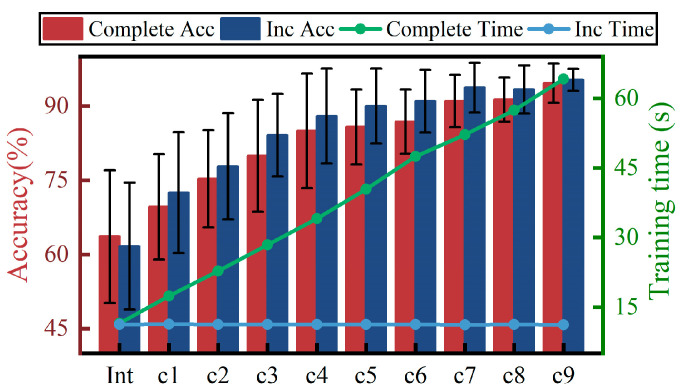
Comparison of training performance using complete data and training using incremental learning, where the bar graph shows the recognition rate and the dotted line graph shows the training time.

Figure 12 shows the performance comparison between the model trained with complete data and that trained with incremental learning. The results show that both methods exhibit a trend of an increasing recognition rate with increasing data. In terms of the overall recognition accuracy, training with incremental learning performed slightly better than training with complete data (*p* < 0.05), reaching a 95.5% recognition rate after the ninth increment, which is about 1% higher than the 94.6% achieved by training with all data. In terms of training time, since the sample size used for incremental learning training is essentially constant, the training time is stable at around 10 s. In contrast, when training with full data, the training time increases as the sample size increases, reaching 64 s after nine increments, and continues to increase. Overall, the training with incremental learning showed superior performance over the training with complete data.

### 4.3. Class Incremental Analysis

This subsection presents the class incremental results for the intertemporal recognition task. In the incremental learning process, one class of actions is selected and set as the incremental action, the initial model is trained from the dataset of the remaining six classes of actions, the incremental dataset consists of the incremental action dataset, the incremental training is performed sequentially according to the time span of the test set, and the training result consists of the representative 1- and 12-day sets as the test set only.

**Figure 13:**
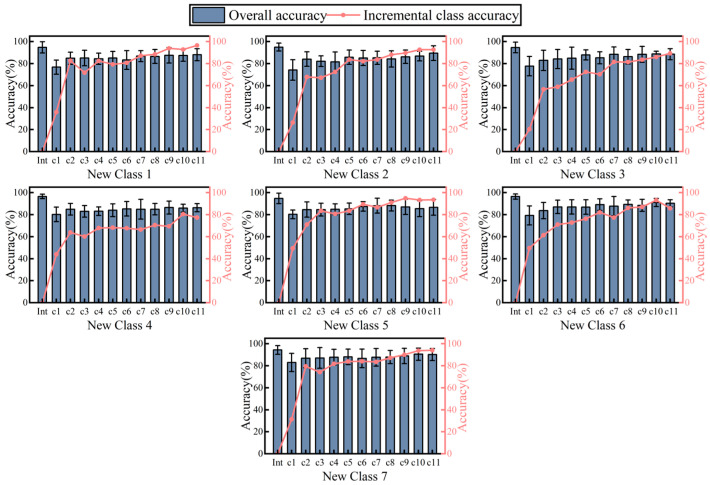
Summary graph of incremental results for different classes, where the bar chart represents the overall recognition rate and the dotted line chart represents the recognition rate of the incremental classes.

The class increment results are shown in Figure 13, where different subplots represent the incremental results for different classes. The results show that the overall recognition accuracy of the model decreases after new classes of data for incremental training are added, and that the overall recognition rate of the model shows a rebound trend as more data are read by the incremental training, reaching an average of 88.6% after completing the incremental training 11 times. The results obtained from the incremental training of different classes are different, as shown in the seven subgraphs. The best result was obtained by the addition of Class 7 (CF), which finally reaches a recognition rate of 90.2%, and the worst was obtained by Class 4 (WUD), which finally reaches a recognition rate of 86.4%. This difference is mainly due to the difference in the way the force is applied: the CF movement relies mainly on the finger force, which is significantly different from other wrist movements, while the way the force is applied in the WUD movement is more similar to in other movements, making it difficult to distinguish in the incremental category. A comparison of the recognition rates of the initial and final models shows that there is a decrease in the recognition performance of the model after the implementation of the class increment, with an average recognition rate of 95.2% before the increment compared to 88.6% after the increment.

## 5. Discussion

In this study, a gesture recognition framework based on DenseNet and replayed incremental learning is proposed to complete the task of gesture action recognition across time using one-dimensional sEMG data as the input. In the selection of CNN models, it is important to note that high-performance models can improve the final recognition rate of the framework. This study compares four models, AlexNet, GoogleNet, ResNet, and DenseNet [39], which are the ones have good performances, including their recognition ability in single-day and cross-day analysis, training time, and model parameters; the detailed results are shown in Table 4. All training details such as the dataset loaded during training, learning rate, number of batches, number of training rounds, etc., are the same for the four models. Finally, the DenseNet model, which has the best performance in each project, is selected as the CNN model for the framework.

The gesture recognition framework proposed in this study overcomes the undesired effects of electrode displacement and time-varying characteristics of the signal in a cross-time task. The recognition accuracy of the model shows a trend of increasing in increments, reaching a recognition rate of 96.5% after several increments. Compared to the results of training with full data, the framework demonstrates superior recognition accuracy. At the same time, it has a great advantage in terms of training time, and can thus be used to solve the instability in the cross-time task with little training cost and storage space. This study also accomplished the class incremental recognition task in the proposed framework. The model still maintains good performance and achieves a recognition rate of 88.6% after multiple increments. However, this result still shows a decrease in the performance of the model compared to the model before class incrementation was performed. This phenomenon is similar to that seen in the results shown in other studies [27,28], where the performance degradation of the model is inevitable in class-increment scenarios, as the task complexity increases with the number of gestures, different from the task-increment and domain-increment scenarios.

The traditional weight in incremental learning is the ratio of the number in the old task to the number in the new task. For the sEMG application scenario, observing Figure 9 shows that the validity of the data decreases as the time span increases, while the new data are more valid due to the shorter time span, meaning that higher weights should be used. In this study, we limit the weight of the new data so that the weight maintains a certain proportion and is not too low. In fact, this weight should be dynamic, and the weight for data with a longer time span should be reduced. In this study, due to the short time span of the experimental design, the use of dynamic weights had a minimal effect on the results. In future research, experiments with longer time spans will be designed and this dynamic weighting may be refined to improve the performance of the model under large time spans, further investigating the problem of catastrophic forgetting in class increments.

## 6. Conclusions

Aiming to address the instability of sEMG signals in the cross-time recognition task, this study investigates the effects of electrode displacement and time-varying characteristics on the recognition accuracy of the studied model and proposes a replay incremental learning framework based on the DenseNet. The framework learns features from non-synchronous data with different time spans in a step-wise incremental manner and achieves an average recognition rate of 96.5% on eight subjects. This study also proposes the use of the DBSCAN clustering algorithm to select representative samples for updating the replay dataset, which outperforms the three sample selection methods, namely uniform selection, random selection, and intermediate selection, in the case of fewer samples. At the same time, the incrementation of the action class is implemented on the framework, which facilitates the action increment to be customized according to the requirements and increases the flexibility of the sEMG signal-based action pattern recognition technique in practical applications.

## Figures and Tables

**Table 1:** The application of the incremental learning method in recent years.

Research Group	Year	Method	Application Areas
Wong et al. [35]	2022	Regularization	Handling noise
Hua et al. [28]	2023	Replay	Gesture classification
Shi et al. [34]	2023	Regularization	Image classification
Alaeiyan et al. [36]	2024	Regularization	Navigation
Thandiackal et al. [37]	2024	Replay	Image classification

**Table 2:** Summary of accuracy in cross-day analysis.

Days	1	2	3	4	5	6	7	8	9	10	11	12
1	-	68%	53%	51%	52%	49%	47%	49%	50%	49%	50%	48%
2	59%	-	68%	75%	62%	58%	52%	48%	60%	57%	59%	55%
3	54%	73%	-	68%	67%	65%	63%	64%	65%	68%	62%	57%
4	53%	79%	72%	-	69%	78%	63%	67%	69%	71%	68%	70%
5	46%	67%	64%	70%	-	66%	62%	62%	66%	71%	62%	65%
6	47%	66%	68%	75%	70%	-	62%	66%	71%	70%	65%	68%
7	44%	62%	61%	62%	68%	62%	-	64%	65%	65%	56%	64%
8	45%	59%	62%	69%	67%	72%	69%	-	76%	69%	74%	73%
9	43%	62%	61%	68%	69%	71%	60%	70%	-	74%	76%	71%
10	50%	61%	70%	69%	72%	71%	66%	73%	79%	-	80%	76%
11	48%	65%	63%	66%	67%	65%	58%	72%	79%	81%	-	76%
12	43%	60%	57%	68%	68%	69%	66%	70%	74%	72%	76%	-

**Table 3:** Incremental learning training strategy.

Test	Train		Incremental Number								
			1	2	3	4	5	6	7	8	9
1	12	11	10	9	8	7	6	5	4	3	2
2	12	11	10	9	8	7	6	5	4	3	1
3	12	11	10	9	8	7	6	5	1	4	2
4	12	11	10	9	8	7	1	6	2	5	3
5	12	11	10	9	1	8	2	7	3	6	4
6	12	11	1	10	2	9	3	8	4	7	5
7	12	1	2	11	3	10	4	9	5	8	6
8	1	2	3	4	12	5	11	6	10	7	9
9	1	2	3	4	5	6	12	7	11	8	10
10	1	2	3	4	5	6	7	8	12	9	11
11	1	2	3	4	5	6	7	8	9	10	12
12	1	2	3	4	5	6	7	8	9	10	11

**Table 4:** Performance comparison of different models.

Model	AlexNet	GoogLeNet	ResNet	DenseNet
Single-day accuracy	91.2%	91.3%	99.6%	99.8%
Cross-day accuracy	55.7%	52.2%	61.8%	64.2%
Training time (s)	25.7	16.6	5.8	4.9
Params (M)	175.3	6.2	3.8	0.4

## Data Availability

The original data presented in the study are openly available in FigShare at https://figshare.com/s/5ea2e795b584bd746a81 (accessed on 16 October 2024).

## References

[1] Ashraf H., Shafiq U., Sajjad Q., Waris A., Gilani O., Boutaayamou M., Brüls O. (2023). Variational mode decomposition for surface and intramuscular EMG signal denoising. Biomed. Signal Proces..

[2] Jie J., Liu K.R., Zheng H., Wang B.B., Dai R. (2021). High dimensional feature data reduction of multichannel sEMG for gesture recognition based on double phases PSO. Complex Intell. Syst..

[3] Lv X., Dai C., Liu H., Tian Y., Chen L., Lang Y., Tang R., He J. (2023). Gesture recognition based on sEMG using multi-attention mechanism for remote control. Neural Comput. Appl..

[4] Li Q., Langari R. (2023). Myoelectric human computer interaction using CNN-LSTM neural network for dynamic hand gesture recognition. J. Intell. Fuzzy Syst..

[5] Cho G., Yang W., Lee D., You D., Lee H., Kim S., Lee S., Nam W. (2023). Characterization of signal features for real-time sEMG onset detection. Biomed. Signal. Process..

[6] Bertomeu-Motos A., Ezquerro S., Barios J.A., Catalán J.M., Blanco-Ivorra A., Martínez-Pascual D., Garcia-Aracil N. (2023). Feasibility of an Intelligent Home-Based Neurorehabilitation System for Upper Extremity Mobility Assessment. IEEE Sens. J..

[7] Toledo-Pérez D.C., Aviles M., Gómez-Loenzo R.A., Rodríguez-Reséndiz J. (2024). Feature set to sEMG classification obtained with Fisher Score. IEEE Access.

[8] Aviles M., Sánchez-Reyes L.M., Fuentes-Aguilar R.Q., Toledo-Pérez D.C., Rodríguez-Reséndiz J. (2022). A novel methodology for classifying EMG movements based on SVM and genetic algorithms. Micromachines.

[9] Prabhavathy T., Elumalai V.K., Balaji E. (2024). Hand gesture classification framework leveraging the entropy features from sEMG signals and VMD augmented multi-class SVM. Expert Syst. Appl..

[10] Wen R., Wang Q., Li Z. (2021). Human hand movement recognition using infinite hidden Markov model based sEMG classification. Biomed. Signal Process..

[11] Hye N.M., Hany U., Chakravarty S., Akter L., Ahmed I. (2023). Artificial Intelligence for sEMG-based Muscular Movement Recognition for Hand Prosthesis. IEEE Access.

[12] Wu D., Tian P., Zhang S., Wang Q., Yu K., Wang Y., Gao Z., Huang L., Li X., Zhai X. (2024). A Surface Electromyography sEMG System Applied for Grip Force Monitoring. Sensors.

[13] Xue J., Sun Z., Duan F., Caiafa C.F., Solé-Casals J. (2023). Underwater sEMG-based recognition of hand gestures using tensor decomposition. Pattern Recognit. Lett..

[14] Zhang Y., Xia C.M., Cao G.S., Zhao T.T., Zhao Y.P. (2024). Pattern recognition of hand movements based on multi-channel mechanomyography in the condition of one-time collection and sensor doffing and donning. Biomed. Signal Process..

[15] Xiong B., Chen W., Niu Y., Gan Z., Mao G., Xu Y. (2023). A Global and Local Feature fused CNN architecture for the sEMG-based hand gesture recognition. Comput. Biol. Med..

[16] Zhang Z., Shen Q., Wang Y. (2024). Electromyographic hand gesture recognition using convolutional neural network with multi-attention. Biomed. Signal Process..

[17] Qureshi M.F., Mushtaq Z., Rehman M.Z.U., Kamavuako E.N. (2023). E2cnn, An efficient concatenated cnn for classification of surface emg extracted from upper limb. IEEE Sens. J..

[18] Chai Y., Liu K., Li C., Sun Z., Jin L., Shi T. (2021). A novel method based on long short term memory network and discrete-time zeroing neural algorithm for upper-limb continuous estimation using sEMG signals. Biomed. Signal Process..

[19] Sun X., Liu Y., Niu H. (2023). Continuous Gesture Recognition and Force Estimation using sEMG signal. IEEE Access.

[20] Lu Y., Wang H., Zhou B., Wei C., Xu S. (2022). Continuous and simultaneous estimation of lower limb multi-joint angles from sEMG signals based on stacked convolutional and LSTM models. Expert Syst. Appl..

[21] Ma X., Liu Y., Song Q., Wang C. (2020). Continuous estimation of knee joint angle based on surface electromyography using a long short-term memory neural network and time-advanced feature. Sensors.

[22] Wu Y., Liang S., Ma Y., Li B. (2024). Prediction and classification of sEMG-based pinch force between different fingers. Expert Syst. Appl..

[23] Fu Y.L., Song W., Xu W., Lin J., Nian X. (2024). Feature recognition in multiple CNNs using sEMG images from a prototype comfort test. Comput. Methods Programs Biomed..

[24] He J., Sheng X., Zhu X., Jiang N. (2019). A novel framework based on position verification for robust myoelectric control against sensor shift. IEEE Sens. J..

[25] Refai M.I.M., Moya-Esteban A., Sartori M. (2024). Electromyography-driven musculoskeletal models with time-varying fatigue dynamics improve lumbosacral joint moments during lifting. J. Biomech..

[26] Hargrove L., Englehart K., Hudgins B. The effect of electrode displacements on pattern recognition based myoelectric control. Proceedings of the 2006 International Conference of the IEEE Engineering in Medicine and Biology Society.

[27] Van de Ven G.M., Tuytelaars T., Tolias A.S. (2022). Three types of incremental learning. Nat. Mach. Intell..

[28] Hua S., Wang C., Lam H.K., Wen S. (2023). An incremental learning method with hybrid data over/down-sampling for sEMG-based gesture classification. Biomed. Signal Process..

[29] Atzori M., Gijsberts A., Castellini C., Caputo B., Hager A.G.M., Elsig S., Giatsidis G., Bassetto F., Müller H. (2014). Electromyography data for non-invasive naturally-controlled robotic hand prostheses. Sci. Data.

[30] Zhao Z., Guo W., Xu Y., Sheng X. (2024). A biosignal quality assessment framework for high-density sEMG decomposition. Biomed. Signal Process..

[31] Zhang Y., Cao G.S., Sun M.X., Zhao B.G., Wu Q., Xia C.M. (2024). Mechanomyography signals pattern recognition in hand movements using swarm intelligence algorithm optimized support vector machine based on acceleration sensors. Med. Eng. Phys..

[32] Ganiga R., Muralikrishna S.N., Choi W., Pan S. (2024). ResNet1D-Based Personal Identification with Multi-Session Surface Electromyography for Electronic Health Record Integration. Sensors.

[33] Jahmunah V., Ng E.Y.K., Tan R.S., Oh S.L., Acharya U.R. (2023). Uncertainty quantification in DenseNet model using myocardial infarction ECG signals. Comput. Methods Programs Biomed..

[34] Shi Y., Shi D., Qiao Z., Wang Z., Zhang Y., Yang S., Qiu C. (2023). Multi-granularity knowledge distillation and prototype consistency regularization for class-incremental learning. Neural Netw..

[35] Wong H.T., Leung H.C., Leung C.S., Wong E. (2022). Noise/fault aware regularization for incremental learning in extreme learning machines. Neurocomputing.

[36] Alaeiyan H., Mosavi M.R., Ayatollahi A. (2024). Improving the performance of GPS/INS integration during GPS outage with incremental regularized LSTM learning. Alex. Eng. J..

[37] Thandiackal K., Portenier T., Giovannini A., Gabrani M., Goksel O. (2024). Generative feature-driven image replay for continual learning. Image Vision Comput..

[38] Li X., Liang S., Yan S., Ryu J.S., Wu Y. (2023). Adaptive detection of Ahead-sEMG based on short-time energy of local-detail difference and recognition in advance of upper-limb movements. Biomed. Signal Process..

[39] Zhang Y., Li T., Zhang X., Xia C., Zhou J., Sun M. (2024). An end-to-end hand action recognition framework based on cross-time mechanomyography signals. Complex Intell. Syst..

